# A bioinspired pseudopeptide-based intracellular delivery platform enhances the cytotoxicity of a ribosome-inactivating protein through multiple death pathways†

**DOI:** 10.1039/d4bm00600c

**Published:** 2024-08-13

**Authors:** Gabriella Morrison, Nicole Henry, Michal Kopytynski, Rongjun Chen

**Affiliations:** a Department of Chemical Engineering, Imperial College London South Kensington Campus London SW7 2AZ UK rongjun.chen@imperial.ac.uk +44 (0)20 7594 2070

## Abstract

Saporin is a 28 621 Da protein and plant toxin possessing rRNA *N*-glycosidase activity. Due to its potent ribosome-inactivating ability, saporin is commonly studied as an anticancer agent. However, its enzymatic activity is greatly hindered by its poor plasma membrane permeability. To overcome this barrier, we used a bioinspired intracellular delivery platform based on the pH-responsive pseudopeptide, poly(l-lysine isophthalamide) grafted with l-phenylalanine at a stoichiometric molar percentage of 50% (PP50). PP50 was co-incubated with saporin (PP50/saporin) in a mildly acidic pH environment to aid intracellular delivery and increase saporin's therapeutic potential. We demonstrated that PP50 greatly enhanced the cytotoxicity of saporin in the 2D monolayer of A549 cells and 3D A549 multicellular spheroids whilst remaining non-toxic when administered alone. To elucidate the mechanism of cell death, we assessed the activation of caspases, the inhibition of protein synthesis, the onset of apoptosis and the mechanism of PP50/saporin entry. Inhibition of protein synthesis and activation of caspases 3/7, 8 and 9 were found to occur before the onset of apoptosis and cell death. PP50/saporin was also shown to rely on micropinocytosis and caveolae-mediated endocytosis for cell entry. In addition, fluorescein isothiocyanate-labelled saporin (FITC-saporin) was localized within the cytoplasm and nuclei when delivered with Cyanine5-labelled PP50 (Cy5-PP50). Taken together, this suggests that multiple pathways are triggered to initiate apoptosis and cell death in cells treated with PP50/saporin. Therefore, these results make PP50 a potential intracellular delivery platform for the internalization of protein therapeutics.

## Abstract 2

A bioinspired intracellular delivery platform based on a pH-responsive pseudopeptide enhances the cytotoxicity of an anticancer protein in 2D monolayer of cells and 3D multicellular spheroids through multiple death pathways.

## Graphical abstract

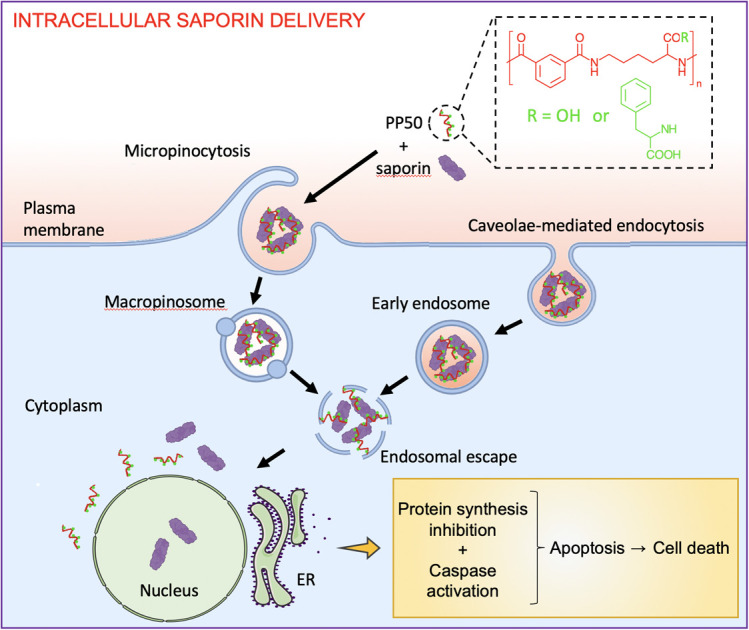

## 1. Introduction

Therapeutic biomacromolecules, such as peptides and proteins, possess biological activity that makes them desirable candidates for study in the field of cancer therapy. These highly specific agents must first bypass the plasma membrane and reach their target site within the cell to exert their biological function.^1–3^ However, the low permeability of cell membranes, coupled with a high chance of succumbing to lysosomal degradation upon entering the endocytosis pathway, remains a barrier for efficient targeted intracellular delivery.^4^

Ribosome-inactivating proteins (RIPs) are a subset of proteins that have gained significant attention in the field of cancer therapy research due to their highly desirable features as potent therapeutics, including toxic *N*-glycosidase activity, high potency at low picomolar range, thermodynamic stability and resistance to high temperatures, denaturation, proteolysis and chemical modifications.^5–8^ Type II RIPs, such as ricin and abrin, contain a B-chain that facilitates binding to the cell surface and fast direct internalization to the cytosol. Comparatively, type I RIPs lack the cell surface binding B-chain, rendering them virtually ineffective unless intracellular delivery is triggered.^9–12^ Saporin is a 28 621 Da type I RIP isolated from the seeds of *Saponaria officinalis*, possessing RNA *N*-β-glycosidase enzymatic activity.^5,9,10^ If given an entry method to bypass the cell membrane, saporin has been reported to deprotonate an adenine residue at position 4324 in the 28S rRNA leading to irreversible inhibition of protein synthesis and cell death *via* apoptosis.^13^ Whilst cell surface binding and internalization are major rate-limiting factors for saporin to exert its biological effect, this distinguishable quality can be exploited to specifically target saporin to cancer cells using intracellular delivery modalities. As such, the use of intracellular delivery agents to improve saporin's intracellular release has attracted much attention.^14–20^ However, a consensus has not been reached regarding the defined method of intracellular saporin delivery and cell death mechanism upon entry.

An anionic pH-responsive amphiphilic pseudopeptide, PP50, has been developed to mimic fusogenic viral peptides by grafting l-phenylalanine at a degree of substitution of 50 mol% of the carboxylic acid groups pendant to the backbone of poly(l-lysine isophthalamide) (PLP).^21–23^ In mildly acidic environments, the protonation of the carboxylic acid groups on the lysine and phenylalanine residues promotes a coil-to-globule conformational change. The predominant hydrophobic regions of the globular structure enable the pseudopeptidic polymer to associate more strongly with the membrane, causing reversible permeabilization of the cell membrane.^24–29^ The metabolite-derived PP50 is biodegradable, unlike other anionic polymers such as vinyl poly(α-alkylacrylic acid) polymers, which have been studied for their applications in cytoplasmic drug delivery.^22^ The anionic PP50 biopolymer is also non-toxic and compatible with a wide range of cell types including cancerous and non-cancerous cells, blood cells and stem cells,^30^ which is a noticeable advantage compared to cationic polymers that are commonly known to be cytotoxic.^31^ Altogether, these qualities render PP50 a suitable intracellular delivery platform and a strong candidate for the delivery of saporin.

**Fig. 1:**
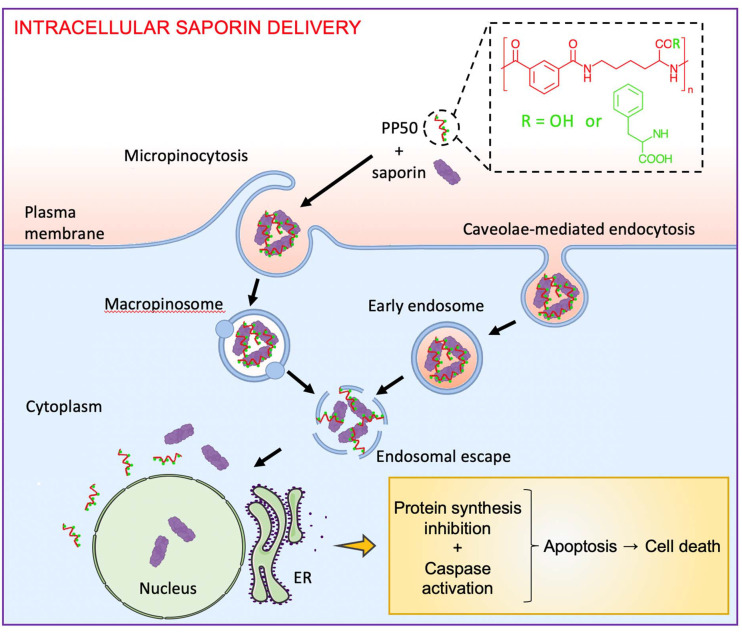
Schematic illustration of the chemical structure of the pH-responsive membrane-permeabilizing pseudopeptide, PP50, and its use for intracellular delivery of saporin for cancer therapy application. PP50/saporin relies on micropinocytosis and caveolae-mediated endocytosis for cell entry, which is followed by endosomal escape. This leads to the inhibition of protein synthesis and activation of caspases 3/7, 8 and 9 before the onset of apoptosis and cell death.

PP50 has recently been shown to facilitate the intracellular delivery of the small molecule trehalose to erythrocytes and osteosarcoma cells for cell preservation^26,29,32,33^ and the pro-apoptotic peptide Bim (*M*_w_ = 2865 Da) to A549 cells for the initiation of cancer cell death through the Caspase 3/7 cascade.^30^ However, the functional delivery of larger proteins in depth has not yet been explored. In the present study, we focused on exploring the ability of PP50 to facilitate the cell entry of saporin (*M*_w_ = 28 621 Da) by co-incubation to enhance its cytotoxicity and further sought to investigate the internalization pathways, 3D penetration in multicellular cancer spheroids and cell death mechanisms initiated (Fig. 1). This work presents PP50 as an excellent delivery platform for potential application in cancer therapy fields.

## 2. Materials and methods

### 2.1. Materials

Saporin from *Saponaria officinalis* seeds (made up of 253 amino acids and with a molecular weight of 28 621 Da), amiloride hydrochloride hydrate, ammonium chloride, nystatin, chlorpromazine hydrochloride, methyl beta-cyclodextrin (MβCD), *N*,*N*′-dicyclohexylcarbodiimide (DCC), Dulbecco's modified Eagle's medium (DMEM), magnesium- and calcium-containing Dulbecco's phosphate-buffered saline (D-PBS), fetal bovine serum (FBS), fluorescein isothiocyanate (FITC), penicillin, trypsin-ethylenediaminetetraacetic acid (EDTA), dimethyl sulfoxide (DMSO), 4-(dimethylamine)pyridine (DMAP), iso-phthaloyl chloride, and Hoechst 33342 were purchased from Sigma-Aldrich (Dorset, UK). Cytochalasin D, the Click-iT® Plus Alexa Fluor™ 488 OPP Protein Synthesis Assay Kit, and the Dead Cell Apoptosis Kit with Annexin V FITC and propidium iodide (PI) were purchased from Thermo Fisher Scientific (Loughborough, UK). Caspase-Glo 3/7 assay, Caspase-Glo 8 assay, Caspase-Glo 9 assay, CellTiter-Glo 2.0 assay, and CellTiter-Glo® 3D assay kits were purchased from Promega (Southampton, UK). Cyanine5 amine was purchased from Abcam (Cambridge, UK). Dimethylformamide (DMF), anhydrous ethanol, acetone, hydrochloric acid, potassium carbonate, sodium hydroxide, chloroform, diethyl ether, and sodium hydrogen carbonate were purchased from VWR (Lutterworth, UK). l-Lysine methyl ester hydrochloride was purchased from Alfa Aesar (Heysham, UK).

### 2.2. Cell culture

A549 adherent epithelial lung carcinoma cells (ATCC) were grown in DMEM supplemented with 10% (v/v) FBS and 100 units per mL penicillin. The A549 cells were trypsinized using trypsin-EDTA and maintained in a humidified incubator at 37 °C with 5% CO_2_.

### 2.3. Synthesis of FITC-saporin

FITC was dissolved in DMSO (1.0 mg mL^−1^). Saporin was dissolved in D-PBS buffer at pH 9.0 (1.0 mg mL^−1^). FITC solution was added dropwise to the saporin solution at the volume ratio of 1 : 1 and stirred at 4 °C in the dark overnight. The mixture was dialyzed against the D-PBS buffer at pH 7.4 for 24 h (MWCO = 12–14 kDa) and lyophilized using a VirTis BenchTop Pro freeze dryer (SP Scientific, USA) before use.

### 2.4. Synthesis of PP50

PLP (*M*_w_ = 35 700, polydispersity = 1.99) was synthesized and then grafted with the hydrophobic amino acid, l-phenylalanine, at a stoichiometric molar percentage of 50% relative to the pendant carboxylic acid groups along the PLP backbone to prepare the pseudopeptidic polymer, PP50 (*M*_w_ = 46 kDa), according to the previously published method.^21^ Briefly, PLP (3 g, 10.87 mmol [COOH]), l-phenylalanine methyl ester hydrochloride (1.17 g, 5.3 mmol [NH_2_]), triethylamine (2.4 molar equivalents to l-phenylalanine) and DMAP (0.6 g) were dissolved in a binary anhydrous DMSO/DMF solvent (1/3 v/v, 60 mL) and stirred at room temperature. DCC (3.36 g, 3 molar equivalents to l-phenylalanine methyl ester hydrochloride) dissolved in anhydrous DMF (20 mL) was added dropwise to the reaction mixture. The reaction was allowed to proceed at room temperature for 60 h. Afterwards, the solution was vacuum filtered to remove solid impurities. NaOH (5 wt%) was dissolved in anhydrous ethanol (28 mL), and the solution was added to the filtrate. The resulting solution was stirred at room temperature for 3 h. The hydrolyzed polymer was then rapidly poured into five volumes of diethyl ether, and the precipitated polymer was collected by vacuum filtration and re-dissolved in dH_2_O. Finally, the polymer was dialyzed against dH_2_O for one week (MWCO = 12–14 kDa). For PP50 in salt form, the pH was adjusted to pH 7.4 with NaOH (0.2 M) before being lyophilized. For PP50 in acidic form, used for H NMR and FTIR analyses, the pH was adjusted to pH 3 with HCl (2 M), and then the polymer precipitate was collected by vacuum filtration and washed three times using dH_2_O before being lyophilized to obtain a fine white powder.

The actual degree of grafting (44.9 mol%) of PP50 was calculated based on the ratio of the integral of 7.13–7.33 ppm to the integral of 7.40–8.75 ppm in its ^1^H NMR spectrum recorded in *d*_6_-DMSO using a 400 MHz NMR spectrometer (Bruker, Germany) (Fig. S1†).

### 2.5. Synthesis of Cy5-PP50

PP50 (100 mg), Cyanine5 amine (3.69 mg) and DMAP (20 mg) were dissolved in DMF (800 μL). DCC (15 mg) was dissolved in DMF (80 μL). The DCC solution was added dropwise to the PP50 solution whilst stirring at room temperature, and the reaction was allowed to proceed for 24 h. The resulting solution was then centrifuged at 9500*g* for 5 min, and the supernatant was dissolved in 25 mL of aqueous sodium bicarbonate (0.1 M). The mixture was dialyzed against dH_2_O (MWCO = 12–14 kDa) for one week and then lyophilized before use.

### 2.6. Preparation of PP50/saporin formulations

The D-PBS solutions at pH 6.5 containing PP50 at a specific concentration of 0.025, 0.1, 0.2, 0.5 or 5 mg mL^−1^ and saporin at varying concentrations were vortexed and then sterile-filtered using 0.22 μm syringe filters. The obtained PP50/saporin formulations were measured by dynamic light scattering (DLS) using a Zetasizer Nano ZS (Malvern, UK) with disposable cuvettes (10 mm in diameter) at a 173° backscatter angle, and used to treat A549 cells or spheroids by co-incubation for various time points.

### 2.7. Cell viability assay

Cell survival was determined using the CellTiter-Glo® 2.0 Cell Viability Assay. A549 cells (5 × 10^3^ cells per well in 100 μL of culture medium) were seeded on a white flat-bottom opaque 96-well plate and incubated overnight. The cells were treated with sterile-filtered D-PBS containing free saporin, PP50 or PP50/saporin at varying concentrations and incubated for varying time durations. The cells were washed three times post-treatment with D-PBS, replenished with DMEM, and then further incubated for 72 h before analysis. After that, the growth medium was replaced with serum-free DMEM (30 μL) plus CellTiter-Glo® 2.0 Cell reagent (30 μL) in each well. The plate was allowed to incubate at room temperature for 10 min to stabilize the luminescent signal before the luminescence was read using a spectrofluorometer (GloMax-Multi Detection System, Promega, USA).

### 2.8. Intracellular distribution

A549 cells (3 × 10^5^ cells per well in 1 mL of culture medium) were seeded in 35 mm glass-bottom dishes (Mattek, USA) and incubated overnight, followed by incubation with free FITC-saporin (0.125 mg mL^−1^), Cy5-PP50 alone (0.2 mg mL^−1^), or Cy5-PP50/FITC-saporin (0.2 mg mL^−1^ Cy5-PP50 and 0.125 mg mL^−1^ FITC-saporin) for 1 h. The cells were washed three times with D-PBS, replenished with DMEM, and then further incubated for 24 h. The cells were stained with Hoechst 33342 (1 μg mL^−1^) and analyzed using a Leica SP8 laser scanning inverted confocal microscope (Zeiss, Germany). The Hoechst 33342, FITC-saporin, and Cy5-PP50 were excited at 405, 488, and 633 nm, respectively. The images were analyzed using FIJI version 2.3.0.

### 2.9. Detection of caspase release

A459 cells (5 × 10^3^ cells per well in 100 μL of culture medium) were seeded and treated as per section 2.6. At 24 h post-treatment on a 96-well plate, the spent medium was replaced with serum-free DMEM (20 μL) plus Caspase-Glo® 3/7, 8 or 9 Assay reagent (20 μL per well) and incubated at room temperature for 1 h. The luminescence was read using a spectrofluorometer (GloMax-Multi Detection System, Promega, USA).

### 2.10. Protein synthesis inhibition assay

A549 cells (5 × 10^3^ cells per well in a 100 μL of culture medium) were seeded on a 96-well cell imaging plate (Eppendorf) overnight. The cells were treated with sterile-filtered D-PBS containing free saporin, PP50 or PP50/saporin at varying concentrations and incubated for 1 h. The cells were washed three times with D-PBS, replenished with DMEM, and further incubated for 24 h before analysis. The commercial Click-iT® Plus Alexa Fluor™ 488 OPP Protein Synthesis Assay Kit was used to analyse protein synthesis inhibition. Treated cells were stained as per the manufacturer's instructions and analyzed using a Leica SP8 laser scanning inverted confocal microscope (Zeiss, Germany). The Hoechst 33342 and Alexa Fluor 488 were excited at 405 and 488 nm, respectively. The images were analyzed using FIJI version 2.3.0.

### 2.11. Apoptosis assay

A549 cells (3 × 10^5^ cells per well in 1 mL of culture medium) were seeded on a 6-well plate and incubated overnight. The cells were incubated with free saporin (1 nM), PP50-0.2 alone (0.2 mg mL^−1^), or PP50-0.2/saporin (0.2 mg mL^−1^ PP50 and 1 nM saporin) for 1 h. After 24 h, 48 h, or 72 h of further incubation, cells were detached with trypsin-EDTA and stained with Annexin V-FITC and PI according to the manufacturer's instructions. The percentages of viable and apoptotic cells were analyzed using a BD LSRFortessa cytometer (BD Biosciences, USA) and visualized using a Leica SP8 laser scanning inverted confocal microscope (Zeiss, Germany). The samples were excited at 488 or 561 nm and the emission was collected in the 530/30 or 610/20 band, respectively. The results were analyzed using FlowJo v10.

### 2.12. Endocytosis inhibition

A549 cells (5 × 10^3^ cells per well in 100 μL of culture medium) were seeded on a white flat-bottom opaque 96-well plate and incubated overnight. The cells were pre-treated with the serum-free DMEM containing cytochalasin D (2.5 μM), chlorpromazine (3 μg mL^−1^), amiloride chloride (30 μM), MβCD (1.5 mg mL^−1^), nystatin (10 μg mL^−1^) or ammonium chloride (100 μM) for 30 min. The spent medium was replaced with sterilized D-PBS containing free saporin, PP50-0.2 alone, or PP50-0.2/saporin with corresponding inhibitors for 1 h. The cells were washed three times with D-PBS, replenished with DMEM, and further incubated for 72 h before analysis with the CellTiter-Glo® 2.0 Cell Viability Assay as described above. Controls were incubated with corresponding inhibitors only.

### 2.13. 3D penetration in multicellular cancer spheroids

A549 cells (2.5 × 10^3^ cells per well in 100 μL of culture medium) were seeded on 96-well ultra-low attachment spheroid microplates (Corning) and cultured in an incubator for 3 days. The spheroids which formed at the bottom of the wells were treated with sterilized D-PBS containing free saporin (300 nM), PP50-5 alone (5 mg mL^−1^), or PP50-5/saporin (5 mg mL^−1^ PP50 and 300 nM saporin) and incubated for 1 h. The cells were washed with D-PBS and replenished with fresh DMEM. After 72 h of further incubation, 50 μL of the CellTiter-Glo® 3D reagent was added to 50 μL of medium containing cells. The contents were mixed vigorously on a plate shaker at 300 rpm for 5 min to induce lysis, and the plate was allowed to incubate at room temperature for a further 25 min before measurement of the luminescence on a spectrofluorometer (GloMax-Multi Detection System, Promega, USA). The cell viability of the spheroids stained with Calcein AM (10 μM) was visualized using a Leica SP8 laser scanning inverted confocal microscope (Zeiss, Germany).

### 2.14. Statistical analysis

Statistical analysis was performed using GraphPad Prism version 9.2.0. Where appropriate, Student's *t*-tests and ANOVA with Tukey's multiple comparison tests were performed. Statistical significance was determined as *P ≤ 0.05, **P ≤ 0.01, ***P ≤ 0.001 and ****P ≤ 0.0001.

## 3. Results

### 3.1. PP50 delivery enhances the cytotoxicity of saporin

The promising enzymatic activity of ribosome-inactivating proteins, such as saporin, after they have crossed the plasma membrane, provides incredible opportunities for cancer therapy.^5^ We have previously shown that the PP50-mediated intracellular delivery of macromolecules is more efficient in mildly acidic pH environments compared to physiological pH.^30^ Consequently, in this study, the PP50-mediated delivery of saporin was investigated using a mildly acidic environment of pH 6.5, which is characteristic of acidic tumor microenvironment.^34,35^

Saporin was co-incubated with PP50 at 0.025 mg mL^−1^ (PP50-0.025/saporin), 0.1 mg mL^−1^ (PP50-0.1/saporin), 0.2 mg mL^−1^ (PP50-0.2/saporin) and 0.5 mg mL^−1^ (PP50-0.5/saporin), respectively. The interaction between PP50 and saporin in an aqueous solution was examined using DLS. As shown in Fig. S2,† like PP50-0.5 alone, PP50-0.5/saporin, which is a mixture of two components, demonstrates a single size distribution. The mean size of PP50-0.5/saporin (45.5 ± 11.5 nm) was slightly larger than that of PP50-0.5 on its own (39.2 ± 8.1 nm), but with no significant difference. The results suggest that PP50 and saporin could interact by complexation subsequent to mixing.

**Fig. 2:**
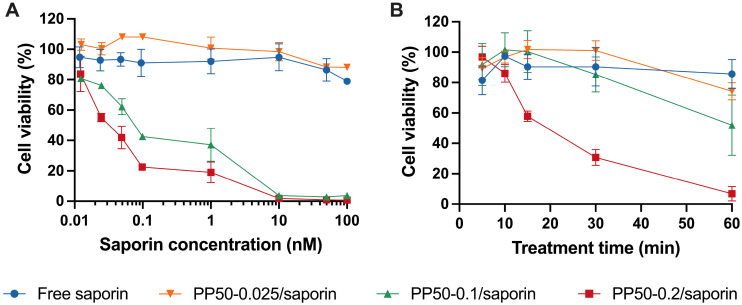
Effects of saporin on the viability of A549 cells in the presence or absence of PP50 at varying concentrations of 0.025, 0.1 and 0.2 mg mL^−1^. Cell viability was evaluated (A) after 1 h of treatment with the indicated concentrations of saporin or (B) in the time-course treatment with 1 nM saporin, plus 72 h of further incubation in DMEM. Mean results ± SD (*n* = 4).

First, to assess the ability of the PP50 delivery platform to deliver saporin into A549 cells, the cells were treated with PP50-0.025/saporin, PP50-0.1/saporin, and PP50-0.2/saporin, respectively, for 1 h, and then were allowed to grow for 72 h before analysis using CellTitre-Glo 2.0® assay. As a control, the dose-dependent cytotoxicity of saporin in its free form (free saporin) was compared. As shown in Fig. 2A, the half maximal effective concentration (EC50), here defined as the concentration of saporin that reduces the cell viability by half, of free saporin and PP50-0.025/saporin remained above 100 nM. In comparison, the increased PP50 concentration in the PP50-0.1/saporin and PP50-0.2/saporin samples resulted in substantially higher cell death corresponding to the decreased EC50 values by at least three orders of magnitude to 0.085 nM and 0.036 nM, respectively. PP50 alone did not show any toxicity to A549 cells (Fig. S3†), which is in good agreement with previous reports of the favorable cellular biosafety of PP50 at workable concentrations and the polymer family it belongs to.^24–26,28–30,32,33^

In treatment time-course experiments (Fig. 2B) using 1 nM saporin, only 22 min of treatment time was required for PP50-0.2/saporin to inhibit cell viability by half, illustrating the fast and efficiency PP50-mediated delivery to enhance the cytotoxicity of saporin.

### 3.2. Saporin delivery activates multiple caspase pathways

**Fig. 3:**
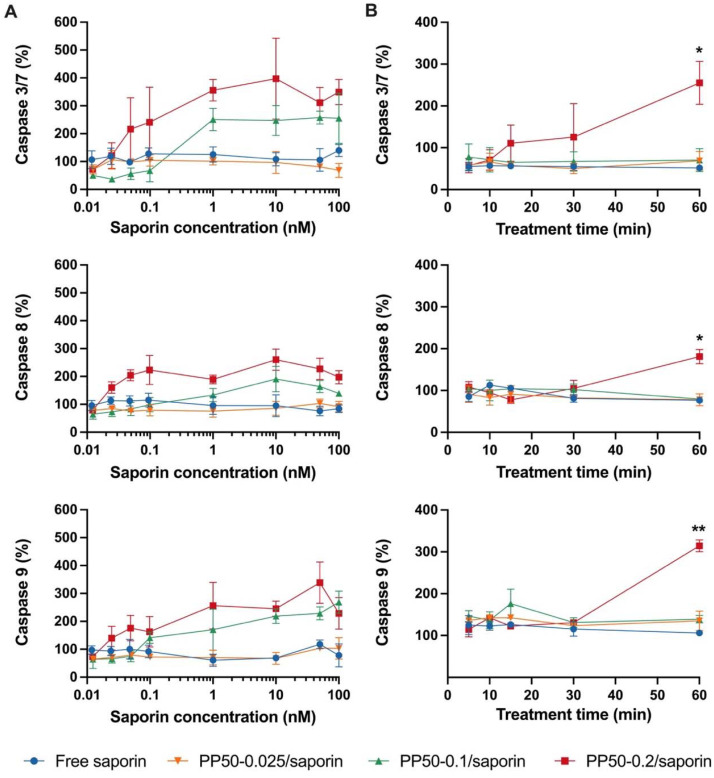
Caspase activation in A549 cells exposed to saporin in the presence or absence of PP50 at varying concentrations of 0.025, 0.1 and 0.2 mg mL^−1^. Caspase 3/7, 8 and 9 activation was evaluated (A) after 1 h of treatment with the indicated concentrations of saporin or (B) in the time-course treatment with 1 nM saporin, followed by 24 h of further incubation in DMEM. Caspase activity is expressed as the percentage of control values obtained from A549 cells grown in the absence of saporin. Mean results ± SD (*n* = 3). *P < 0.05 and **P < 0.01.

Next, we investigated the pathways involved in the cell death mechanism induced by saporin. In our experiments, the ability of saporin to activate the caspase 9 intrinsic pathway, caspase 8 extrinsic pathway and effector caspase 3/7 was studied. Fig. 3A shows that PP50-0.2/saporin and PP50-0.1/saporin were able to activate all three caspase pathways. The caspase 3/7 pathway was activated around 1.25- to 2-fold more than the caspase 8 and 9 pathways. The stronger activation of executioner caspase 3/7 may have been due to the downstream effects of initiator caspases 8 and 9, both converging into the caspase 3/7 pathway.^36^ As a comparison, free saporin and PP50-0.025/saporin produced negligible activation of all three caspase pathways.

Treatment time-dependent studies, using a low concentration of 1 nM saporin, revealed that only in the cells treated with PP50-0.2/saporin did caspase 3/7, 8, and 9 activation become significantly higher than that of free saporin after 1 h of treatment (Fig. 3B).

### 3.3. PP50-0.2/saporin delivery platform inhibits protein synthesis

**Fig. 4:**
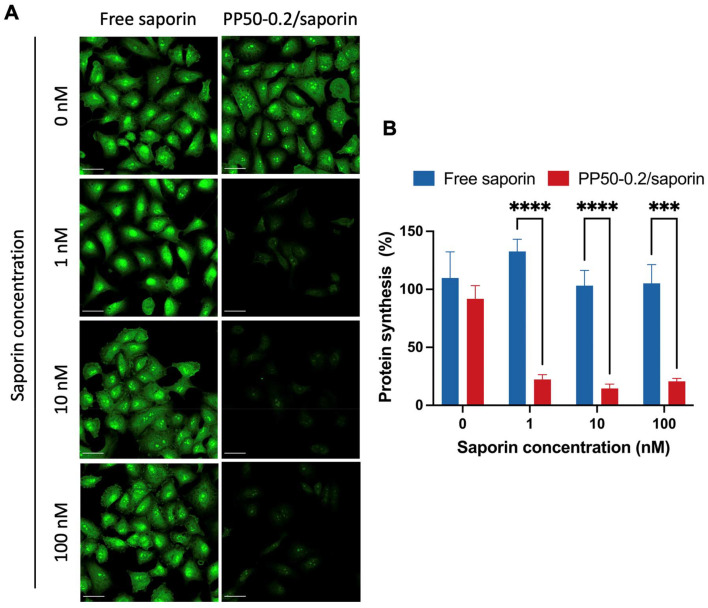
Inhibition of protein synthesis in A549 cells exposed to saporin in the presence or absence of PP50-0.2 using the Click-iT® Plus OPP assay. (A) Confocal images illustrating nascent protein synthesis as detected by Alexa Fluor 488 signal. Protein synthesis inhibition was evaluated after 1 h of treatment with free saporin or PP50-0.2/saporin samples plus 24 h of further incubation in DMEM. Scale bar: 45 μm. (B) Quantitative analysis of protein synthesis percentages by measuring the fluorescence intensity in confocal microscpy images *via* FIJI. Mean results ± SD (*n* = 6, one sample is one individual cell). ***P < 0.001 and ****P < 0.0001.

The optimal PP50 concentration of 0.2 mg mL^−1^ (PP50-0.2) for saporin delivery was selected to test the ability of PP50-0.2/saporin to inhibit protein synthesis. The Click-IT Plus OPP assay (Fig. 4A) showed that free saporin and PP50-0.2 only did not inhibit protein synthesis, as confirmed by the bright green fluorescence within the cell cytoplasm and nucleus. Conversely, A549 cells treated with PP50-0.2/saporin at the saporin concentration ranging from 1 to 100 nM all displayed a marked reduction in fluorescence, suggesting successful protein synthesis inhibition post saporin delivery. In addition, quantitative analysis (Fig. 4B) revealed that after 1 h of treatment with PP50-0.2/saporin, protein synthesis inhibition was significantly lower than that of the controls, validating the reports that saporin is a potent protein synthesis inhibitor once it has entered the cell.^5,37^ A two-way ANOVA test showed the significant difference between the absence of saporin (0 nM) and the delivery of saporin with PP50 at any of the saporin concentrations tested (red columns, *p* < 0.0001), while no significant difference was observed between the PP50-0.2/saporin groups with saporin at 1, 10 and 100 nM, respectively.

Saporin and its cytotoxic effects have been extensively studied over the years. The timeframe in which the reduction of protein synthesis or cessation occurs when treating cells with RIPs is several hours. As reported by Polito *et al.*, protein synthesis was still over 20% 24 h after the initial treatment with saporin.^38^ It is also important to note that many researchers have argued that the inhibition of protein synthesis is not the only way in which saporin can lead to cell death.^5^ Bagga *et al.* have reported that saporin can also induce DNA fragmentation and therefore apoptosis.^39^

### 3.4. Apoptotic effect of PP50-0.2/saporin

**Fig. 5:**
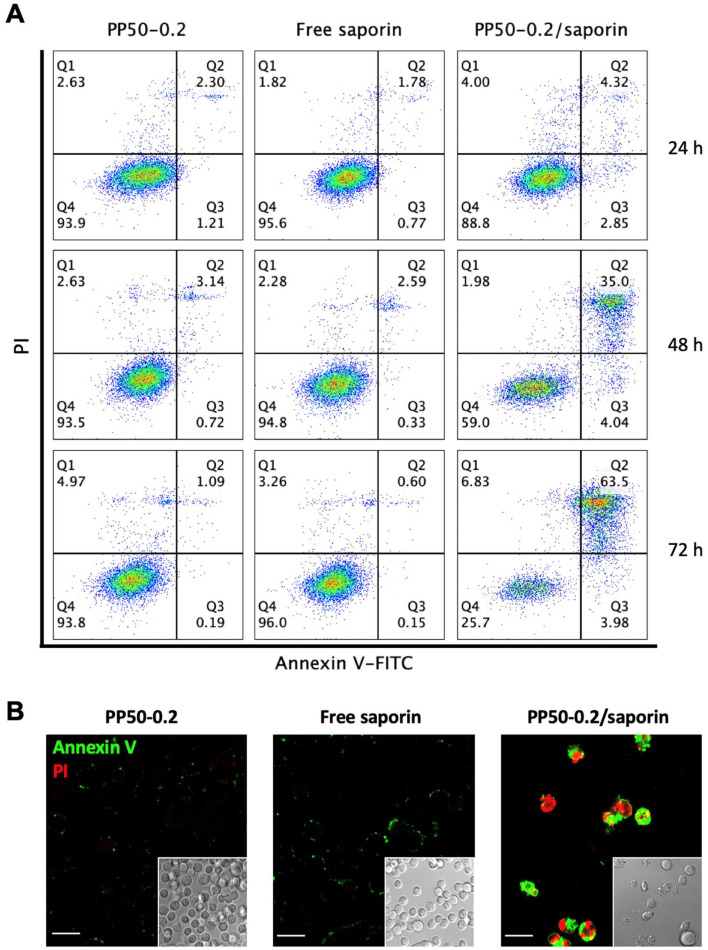
Evaluation of cell health after 1 h of treatment with saporin (1 nM) in the presence or absence of PP50-0.2 plus further incubation in DMEM. (A) Cytofluorometry analysis of Annexin V/PI double staining of A549 cells after 24, 48 and 72 h of further incubation. (B) Fluorescence and phase contrast microscopy analysis of Annexin V/PI double staining of A549 cells after 72 h of further incubation. Scale bar: 20 μm.

To further verify the cell death mechanism, we next analyzed the ability of PP50-0.2/saporin (saporin concentration at 1 nM) to induce apoptosis in A549 cells over time. The cells were treated for 1 h, and cytofluorometric analysis was conducted after 24, 48, or 72 h of further incubation in DMEM. As shown in Fig. 5A, in the free saporin and PP50-0.2 only controls, the majority of cells remained viable (*i.e.*, 93.8% and 96.0% live cells, respectively, as shown in the Q4 quadrant) even after 72 h of further incubation. However, for the PP50-0.2/saporin group, the percentage of live cells (Q4) significantly decreased to 25.7%, while the percentage of PI+/Annexin V+ positive cells significantly increased over the course of 72 h. Confocal microscopy observation of these cells stained with FITC-Annexin V/PI (Fig. 5B and S4†) also showed a double-staining fluorescence pattern indicative of late-stage apoptosis or necrosis in the cell samples treated with PP50-0.2/saporin. Interestingly, there was also a substantial decrease in the number of cells visualized in the PP50-0.2/saporin treatment group (Fig. 5B). These observations are in agreement with the cell viability results taken after 72 h of further incubation, where the cell viability of PP50-0.2/saporin treated with 1 nM saporin decreases to 19.0 ± 6.8% (Fig. 2).

### 3.5. Intracellular distribution and entry mechanism of PP50-0.2/saporin

**Fig. 6:**
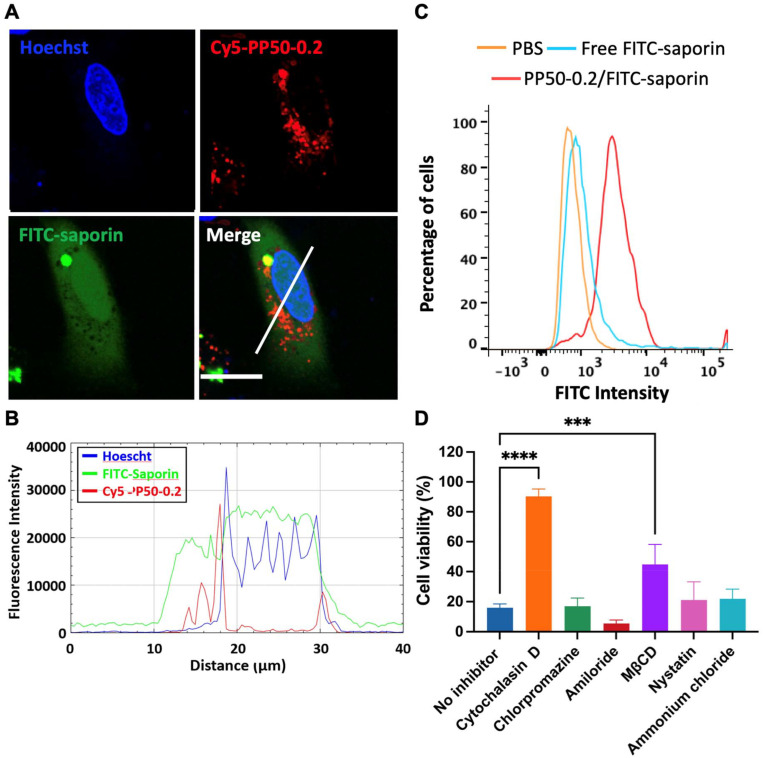
(A) Confocal microscopy images illustrating the intracellular localization in A549 cells after 1 h of treatment with FITC-saporin (125 μg mL^−1^) in the presence of Cy5-PP50-0.2 plus 24 h of further incubation in DMEM. The cell nucleus was counterstained with Hoechst 33342. Scale bar: 20 μm. (B) Fluorescence intensity profiles produced from the cross-section along the white line in image A. (C) Corresponding cytofluorometry analysis. (D) Inhibition of PP50-0.2/saporin cytotoxicity (saporin concentration at 1 nM) in the presence of different inhibitors. Cells were pre-incubated with a specific inhibitor for 30 min. Cell viability was evaluated after 1 h of treatment with the inhibitor plus 72 h of further incubation in DMEM. Mean results ± SD (*n* = 4). ***P < 0.001 and ****P < 0.0001.

To gain an insight into the intracellular distributions of saporin and PP50 in A549 cells, saporin was labelled with green fluorescent FITC, and PP50 was labelled with red fluorescent Cy5. The distribution patterns of Cy5-PP50-0.2 and FITC-saporin (FITC-saporin concentration at 125 μg mL^−1^) after delivery were visualized using confocal microscopy. Fig. 6A and B show that strong green fluorescence diffused throughout the cytoplasm, suggesting the efficient cell entry and endosomal escape of saporin attributed to PP50-mediated delivery. Much of the green fluorescence did not co-localize with the red fluorescence, which is indicative of the dissociation of saporin from PP50 following the polymer-mediated endosomal escape. Interestingly, FITC-saporin, but not Cy5-PP50-0.2, was also localized in the nuclei. As a comparison, there was only marginal green fluorescence and it was shown as green punctate spots when cells were treated with free FITC-saporin only (Fig. S5 and S6†), which is consistent with the report of other researchers that saporin alone has difficulty in entering cells and suffers from endosomal entrapment.^9–12^ Cytofluorometric analysis also showed that the fluorescence intensity of FITC in the cells treated with PP50/FITC-saporin was remarkably higher than that of FITC-saporin only or the D-PBS controls (Fig. 6C).

Subsequently, to further understand the delivery mechanism of PP50-0.2/saporin, A549 cells were pre-treated for 30 min with an inhibitor, such as cytochalasin D, chlorpromazine, amiloride chloride, MCβD, nystatin or ammonium chloride, and treated thereafter with PP50-0.2/saporin in the presence of the corresponding inhibitor for 1 h. Cell viability was determined after 72 h of further incubation. As shown in Fig. 6D, co-treatment with cytochalasin D (an inhibitor of actin polymerization and micropinocytosis) or MCβD (a caveolae-mediated endocytosis inhibitor) significantly reduced the cytotoxic ability of PP50-0.2/saporin. These results also suggest that the intracellular delivery of PP50-0.2/saporin relies predominantly on micropinocytosis compared to caveolae-mediated endocytosis. Co-treatment with cytochalasin D caused almost complete inhibition of the PP50-0.2/saporin delivery with the cell viability remaining at 90.0 ± 4.9%, while co-treatment with MCβD led to only partial inhibition of the PP50-0.2/saporin delivery (reduction of cell viability to 45.0 ± 13.5%). However, treatment with inhibitors alone for the same time period did not affect cell viability (Fig. S7†).

### 3.6. *In vitro* tumor model penetration of saporin in multicellular spheroids

**Fig. 7:**
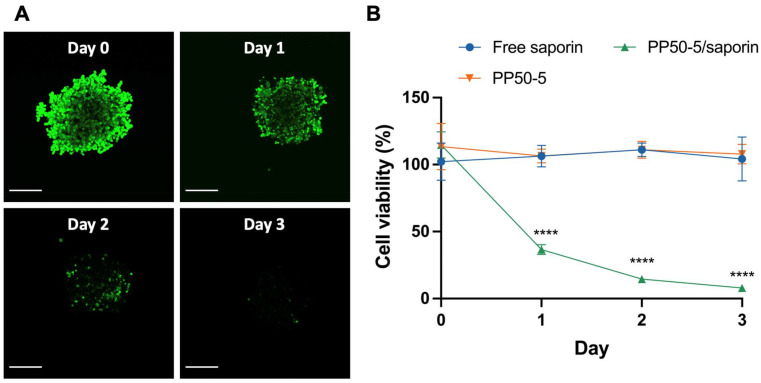
Effects of PP50-5/saporin (saporin concentration at 300 nM) on the cell viability of A549 spheroids over 3 days after 1 h of treatment. (A) Confocal microscopy images of the PP50-5/saporin treated spheroids stained with Calcein AM (scale bar: 200 μm). (B) Cell viabilities after treatment with PP50-5/saporin and the control groups. Mean results ± SD (*n* = 7). ****P < 0.0001.

Based on our *in vitro* findings in 2D monolayer culture of A549 cells, we finally conducted experiments to determine the ability of PP50 to deliver saporin into 3D multicellular A549 spheroids. We have previously shown effective penetration of other l-phenylalanine grafted polymers into tumor spheroid models.^28^ A higher concentration of PP50 at 5 mg mL^−1^ (PP50-5) was used to enable efficient penetration into spheroids. A549 spheroids were grown for 3 days and then treated with PP50-5/saporin (saporin concentration at 300 nM) or the controls for 1 h. Cell viability post-treatment was determined using the CellTiter-Glo® 3D assay, and the live cells were visualized *via* confocal microscopy using Calcein AM over the course of 3 days. Confocal microscopy images in Fig. 7A show a decrease in green fluorescence in the spheroids treated with PP50-5, signifying the number of live cells decreasing from day 0 to day 3. These results were quantitatively confirmed, as shown in Fig. 7B. It can be seen that on day 1, the cell viability of the spheroids treated with PP50-5/saporin decreased to 31.0 ± 3.1%, significantly lower than that for the spheroids treated with D-PBS, PP50-5 only and free saporin (*p* < 0.0001), respectively. In comparison, there was no apparent decrease in cell viability after 3 days with the spheroids treated with free saporin at 300 nM or PP50-5 alone. Conversely, the cell viability of the spheroids treated with PP50-5/saporin continued to decrease to 7.9 ± 2.5% until day 3 (final day).

## 4. Discussion

In this study, we demonstrated that PP50-0.2 allows the efficient delivery of saporin into A549 cells. As a result, these cells undergo a series of biological changes: inhibition of protein synthesis, activation of the caspase pathways and apoptosis, ultimately leading to cell death. Multiple studies have shown that saporin can induce cytotoxicity in the absence of a delivery platform in the range of 10–10^3^ nM saporin,^39–41^ yet the complete mechanism of saporin entry is inconclusive. It has been proposed that saporin may enter *via* the α_2_-macroglobulin receptor^42^ but it was later reported that this receptor might not be necessary for internalization.^41^ Our study has revealed that PP50-0.2/saporin relies predominantly on micropinocytosis for internalization but may also secondarily rely on caveolae-mediated endocytosis for entry. Furthermore, at the concentrations used within this study, we show that free saporin is unable to initiate cell death in A549 cells. It should be noted that direct comparison between studies is challenging due to a difference in the cell lines, cell number and treatment times used.

Previous studies investigating the involvement of caspase pathways and protein synthesis in triggering apoptosis in saporin treated cells have yielded different results. Bolognesi *et al.* showed activation in caspase 3/7, 8 and 9 pathways in L540 cells.^38^ In U937 cells, it was reported that saporin induces apoptosis through the mitochondrial caspase. Saporin increased the activation of caspase 3 and caspase 9 but not caspase 8. Moreover, the onset of apoptosis was not dependent on the inhibition of protein synthesis.^43^ Caspase 3/7 activation was also detected in J774.2 cells treated with saporin.^44^ In the current study, we found that PP50-0.2/saporin was able to activate both the mitochondria-driven intrinsic and extrinsic death receptor pathways 24 h after treatment. This was also accompanied by almost complete inhibition of protein synthesis. Subsequently, an increase in apoptosis and cell death was observed at 48 and 72 h after treatment. Taken together, this suggests that saporin induces apoptosis and cell death through the inhibition of protein synthesis and the intrinsic and extrinsic cascade when co-incubated with our PP50 delivery platform. However, our investigation also revealed the presence of FITC-saporin in the nuclei, which may suggest the involvement of an alternative DNA-dependent apoptotic cell death mechanism to enhance cytotoxicity. In Ca9-22 cells, saporin conjugated with a PAMAM dendrimer carrier was shown to localize in the nuclei after treatment with a photochemical internalization technology.^20^ The presence of saporin in the nuclei has also been observed in HeLa cells after receptor-independent endocytosis.^45^ Further studies are required to elucidate the potential DNA dependent cell death mechanism of saporin delivered *via* PP50.

The use of saporin as an anticancer agent has sparked particular interest, with clinical trials using saporin-based immunotoxins dating back to 1992.^46^ Here, we have demonstrated that PP50-mediated delivery enhances the cytotoxicity of ribosome-inactivating protein saporin in 2D monolayer A549 cells, with PP50-0.2/saporin showing an extremely low EC50 value at 36 pM. In addition, these results can be translated to more biologically relevant 3D multicellular A549 spheroids *in vitro*. Our previous studies have used this pH-responsive polymer to successfully deliver an array of other macromolecular payloads to a wide variety of different cell types. Kopytynski *et al.* investigated PP50 as a versatile delivery platform to enhance the intracellular delivery of macromolecules, including FITC–Dextran of different sizes (average *M*_w_ = 10, 70, 150, and 500 kDa), green fluorescent protein (GFP, *M*_w_ = 26.9 kDa), and the pro-apoptotic peptide Bim (*M*_w_ = 2865 Da) as a functional payload. PP50 facilitated the intracellular delivery of the macromolecular payloads to 9 different cell types, including human lung carcinoma (A549), human cervical cancer (HeLa), human uterus sarcoma (MES-SA) and doxorubicin-resistant MES-SA (MES-SA/Dx5), murine osteoblastic cells (MC 3t3), Chinese hamster ovarian cells (CHO), human B lymphocytes (SUDHL-8), murine macrophage/monocytes (RAW 264.7), and human mesenchymal stem cells (hMSCs).^30^ This shows the potential of PP50 to be used as a platform for the delivery of different cytotoxic proteins or therapeutic molecules to different cell types for cancer therapy applications.

## 5. Conclusions

In conclusion, we have demonstrated the suitability of the anionic pH-responsive amphiphilic pseudopeptide, PP50, in enhancing the cytotoxicity of the cell-impermeable ribosome-inactivating protein saporin. Notably, this platform enabled time-dependent cell death in A549 cells, by which the inhibition of protein synthesis and activation of caspases 8, 9 and 3/7 were found to be critical events prior to the onset of apoptosis and cell death. Internalization of the PP50/saporin construct was mediated by micropinocytosis and caveolae-mediated endocytosis. Furthermore, this platform has demonstrated significant potential for 3D penetration in multicellular cancer spheroids. Beyond the scope of this project, PP50 has shown tremendous potential for targeting proteins to the nucleus, which may be further explored to determine the extent of PP50-mediated biomacromolecule delivery for nuclear targeting. Thus, this intracellular delivery platform holds substantial promise for a wide range of applications in cancer therapy.

## Author contributions

R.C. conceived the project, and guided and supervised the research. R.C., G.M. and M.K. designed the experiments. G.M. completed all the experiments. G.M., N.H. and R.C. analyzed the data and designed the figures; G.M., N.H. and R.C. wrote the paper. All authors have read, commented on, and approved the final version of this paper.

## Data availability

All data relevant to the paper are included in the article and its ESI.† Further details and information can be provided by the corresponding author upon request.

## Conflicts of interest

The authors declare no competing financial interest.

## Supplementary Material

BM-012-D4BM00600C-s001
