# Supplementary material for: A bioinspired pseudopeptide-based intracellular delivery platform enhances the cytotoxicity of a ribosome-inactivating protein through multiple death pathways
Source: Biomater Sci. 2024 Aug 13;12(19):5010–22. doi: 10.1039/d4bm00600c (PMC11342633; doi:10.1039/d4bm00600c)
Supplement: BM-012-D4BM00600C-s001 [file BM-012-D4BM00600C-s001.pdf]

Supplementary Information (SI) for Biomaterials Science.
This journal is © The Royal Society of Chemistry 2024

Supplementary Information

A Bioinspired Pseudopeptide-based Intracellular Delivery Platform Enhances the Cytotoxicity of a Ribosome-inactivating Protein through Multiple Death Pathways

*Gabriella Morrison, Nicole Henry, Michal Kopytynski and Rongjun Chen**

Department of Chemical Engineering, Imperial College London, South Kensington Campus, London, SW7 2AZ, United Kingdom

*Corresponding author:

Email: rongjun.chen@imperial.ac.uk

Tel: +44 (0)20 7594 2070

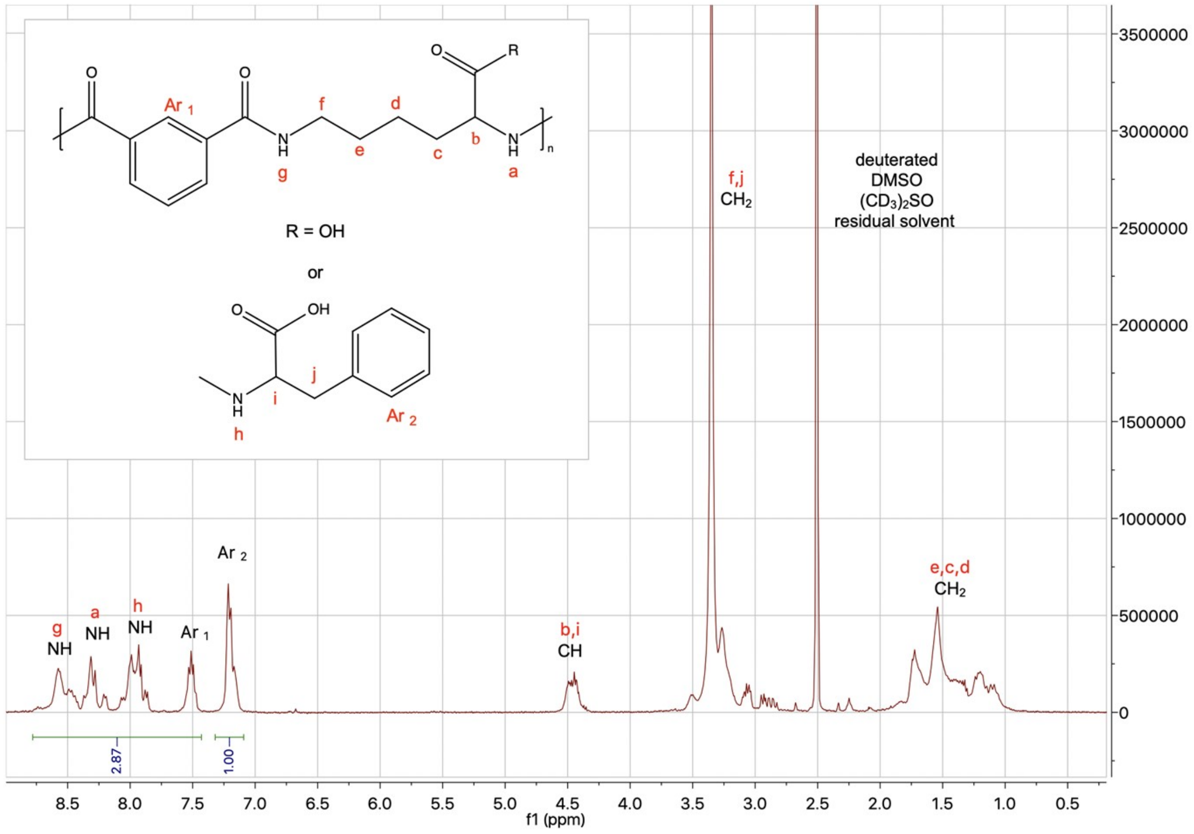

Figure S1. Chemical structure and ^{1}H-NMR spectrum of PP50 in acidic form in d_6-DMSO at room temperature.

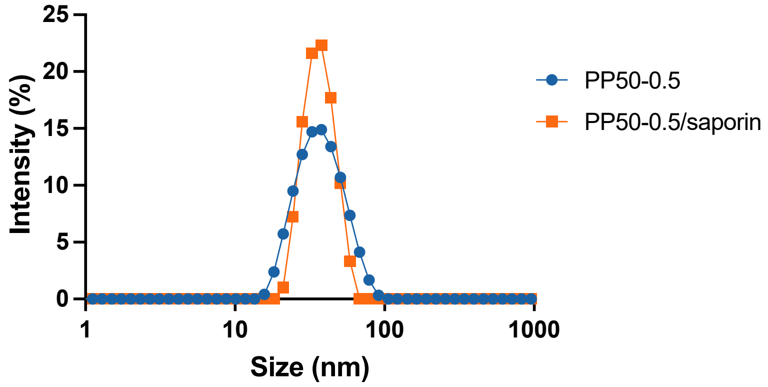

Sample	Diameter (nm)	PDI
PP50-0.5	39.2 ± 8.1	0.210 ± 0.029
PP50-0.5/saporin	45.5 ± 11.5	0.253 ± 0.018

Figure S2. The hydrodynamic size distributions and the summary table of particle diameters of PP50-0.5 and PP50-0.5/saporin at pH 6.5, respectively, measured by dynamic light scattering ([PP50] = 0.5 mg mL^{-1}; [Saporin] = 1 nM). Mean results ± SD (n=3).

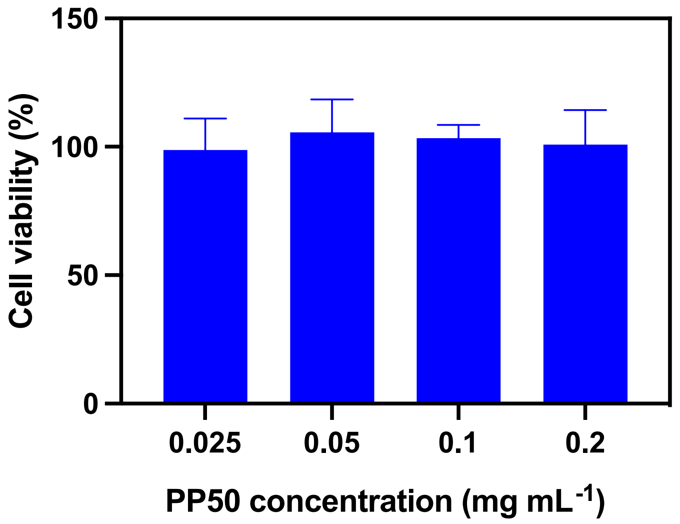

Figure S3. Effects of PP50 at varying concentrations on viability of A549 cells. Cell viability was determined using the CellTiter-Glo® 2.0 Cell Viability Assay after 1 h treatment plus 72 h further incubation in DMEM. Mean results ± SD (n=3).

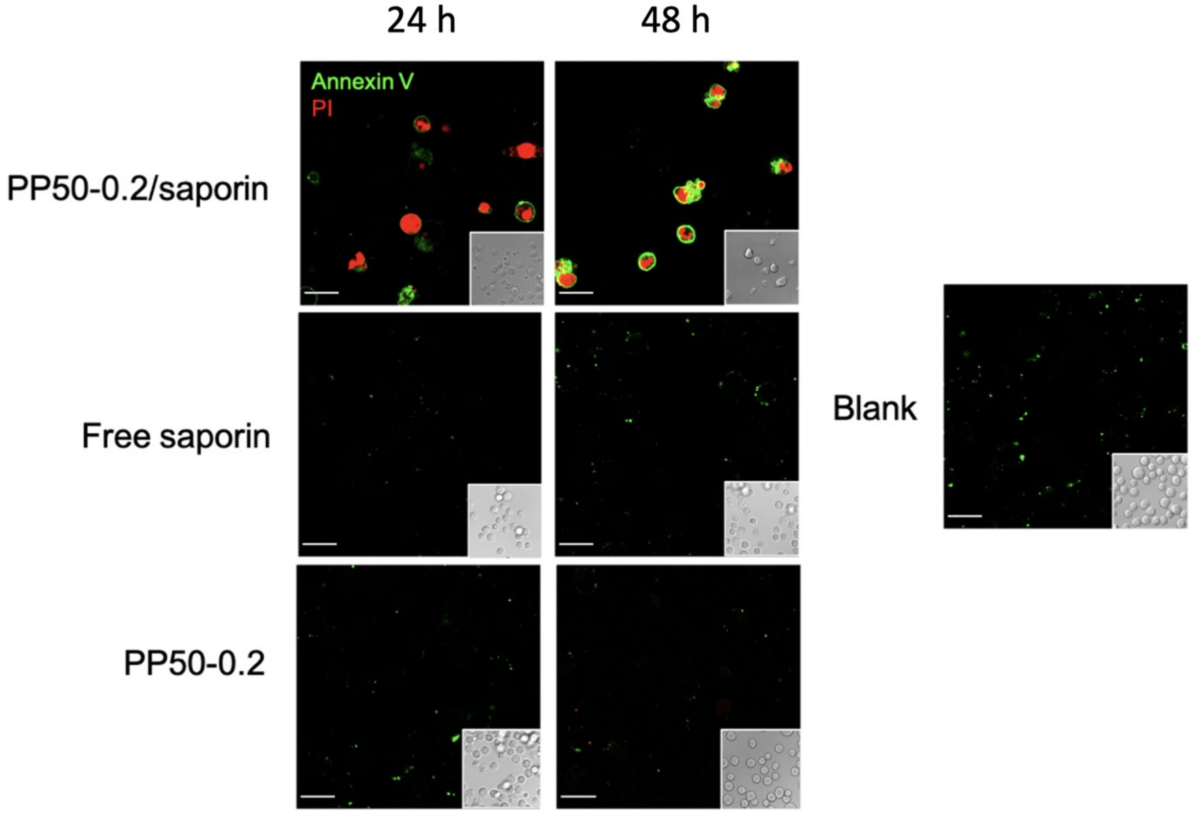

Figure S4. Fluorescence and phase contrast microscopy analysis of Annexin V/PI double staining of A459 cells after exposure to saporin (1 nM) in the presence or absence of PP50-0.2 after 1 h treatment plus 24 or 48 h further incubation in DMEM. Scale bar 20 µm.

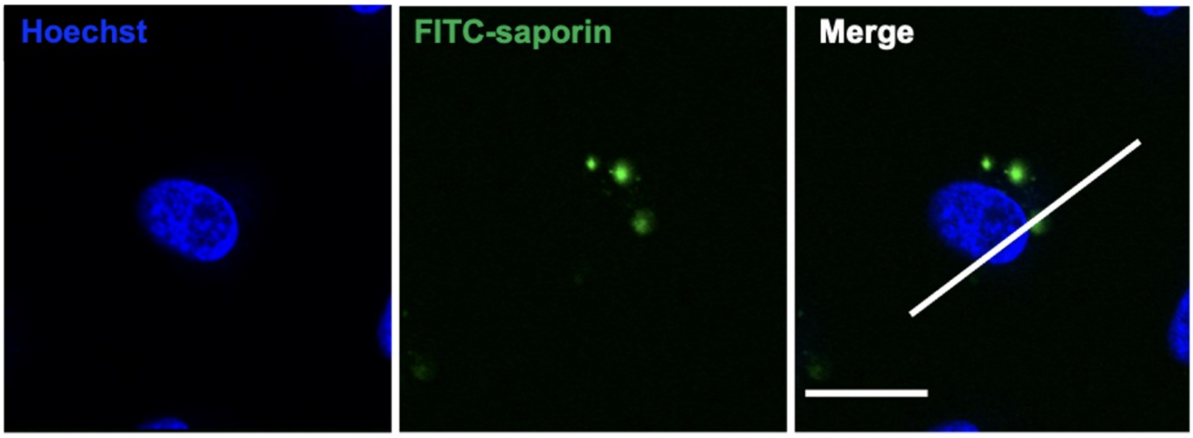

Figure S5. Confocal microscopy images illustrating the intracellular localization in A549 after 1 h treatment with free FITC-saporin (125 µg mL^{-1}) plus 24 h further incubation in DMEM. The cell nucleus was counterstained with Hoechst 33342. Scale bar 20 µm.

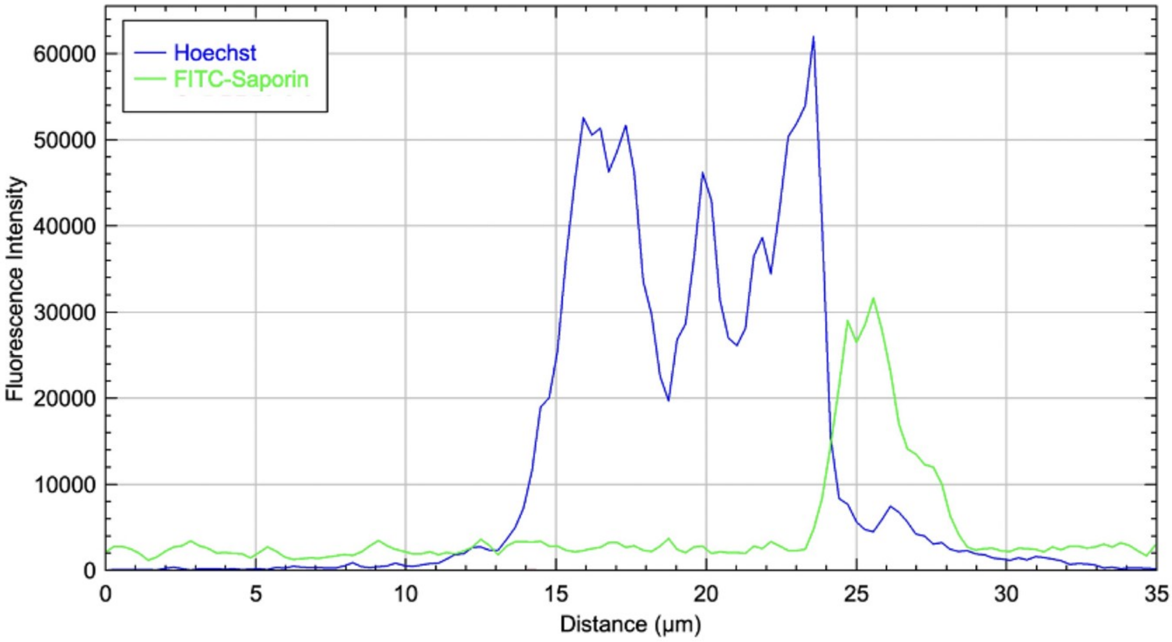

Figure S6. Fluorescence intensity profiles of A549 cells after 1 h treatment with free FITC-saporin (125 µg mL^{-1}) plus 24 h further incubation in DMEM, produced from the cross section along the white line in Figure S4.

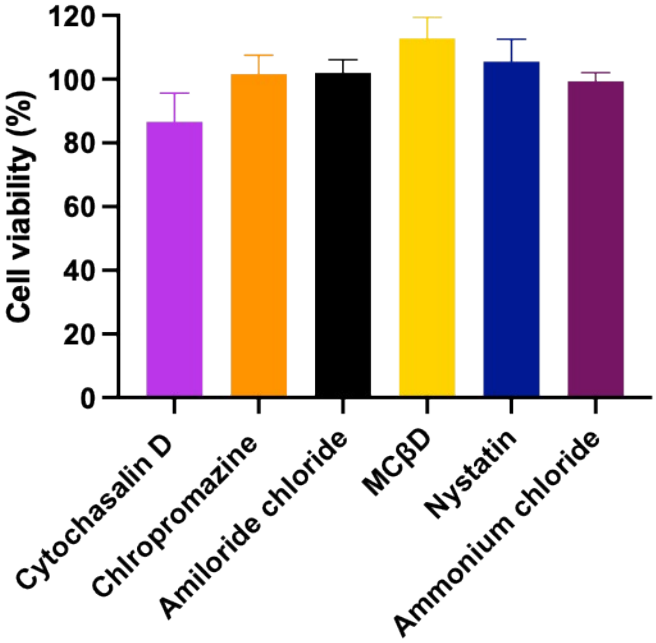

Figure S7. Cell viabilities after inhibitor treatment. A549 cells were pre-incubated with the specific inhibitor for 30 min. Cell viability was determined using the CellTiter-Glo® 2.0 Cell Viability Assay after 1 h treatment with corresponding inhibitor plus 72 h further incubation Mean results ± SD (n = 4).
